# Chromosomal Type II Toxin–Antitoxin Systems May Enhance Bacterial Fitness of a Hybrid Pathogenic *Escherichia coli* Strain Under Stress Conditions

**DOI:** 10.3390/toxins16110469

**Published:** 2024-11-01

**Authors:** Jessika C. A. Silva, Lazaro M. Marques-Neto, Eneas Carvalho, Alejandra M. G. Del Carpio, Camila Henrique, Luciana C. C. Leite, Thais Mitsunari, Waldir P. Elias, Danielle D. Munhoz, Roxane M. F. Piazza

**Affiliations:** 1 Laboratório de Bacteriologia, Instituto Butantan, Avenida Vital Brazil, 1500, São Paulo 05503-900, SP, Brazil; jessika.cristina4@hotmail.com (J.C.A.S.); eneas.carvalho@butantan.gov.br (E.C.); aledani_7@usp.br (A.M.G.D.C.); camilahpp20@gmail.com (C.H.); thaismitsunari@gmail.com (T.M.); waldir.elias@butantan.gov.br (W.P.E.); 2 Laboratório de Desenvolvimento de Vacinas, Instituto Butantan, Avenida Vital Brazil, 1500, São Paulo 05503-900, SP, Brazil; lazaro.neto.esib@esib.butantan.gov.br (L.M.M.-N.); luciana.leite@butantan.gov.br (L.C.C.L.); 3 Instituto de Ensino e Pesquisa Albert Einstein, Rua Comendador Elias Jaffet, 755, São Paulo 05653-000, SP, Brazil

**Keywords:** hybrid strain, toxin–antitoxin type II, gene transcription, stress conditions

## Abstract

The functions of bacterial plasmid-encoded toxin–antitoxin (TA) systems are unambiguous in the sense of controlling cells that fail to inherit a plasmid copy. However, its role in chromosomal copies is contradictory, including stress-response-promoting fitness and antibiotic treatment survival. A hybrid pathogenic *Escherichia coli* strain may have the ability to colonize distinct host niches, facing contrasting stress environments. Herein, we determined the influence of multiple environmental stress factors on the bacterial growth dynamic and expression profile of previously described TA systems present in the chromosome of a hybrid atypical enteropathogenic and extraintestinal *E. coli* strain. Genomic analysis revealed 26 TA loci and the presence of five type II TA systems in the chromosome. Among the tested stress conditions, osmotic and acid stress significantly altered the growth dynamics of the hybrid strain, enhancing the necessary time to reach the stationary phase. Using qPCR analyses, 80% of the studied TA systems were differentially expressed in at least one of the tested conditions, either in the log or in the stationary phase. These data indicate that type II TA systems may contribute to the physiology of pathogenic hybrid strains, enabling their adaptation to different milieus.

## 1. Introduction

Toxin–antitoxin (TA) systems are genetic modules composed of a labile antitoxin (a non-coding RNA or protein) that neutralizes a stable toxin. These systems are composed of two genes typically present in the same operon, encoding the toxin and the antitoxin [1,2,3,4]. There are eight types of TA systems based on the antitoxin nature and mechanism of action. Due to its diversity and number of representatives, the type II TA system is the most characterized, where the formation of a TA complex occurs through a direct protein–protein interaction between the toxin and antitoxin, effectively inhibiting the toxin activity [5,6].

These systems were discovered in the 1980s as modules that were present in plasmids, and, after the characterization of R1 and F plasmids, the “post-segregational killing” (PSK) model was established, referring to its role in controlling cells that fail to inherit a plasmid copy [7,8]. Furthermore, different functions were proposed for plasmid-encoded systems, since they affect important bacterial physiological processes such as DNA replication, translation, cell division, and ATP synthesis [9,10].

DNA sequencing technology and bioinformatic approaches allowed the identification of thousands of TA systems in bacterial chromosomes, and, currently, more than 10,000 TA models are known [11,12]. Despite the well-established functions of plasmid-encoded systems, the proposed roles for their chromosomal counterparts are still inconsistent, being largely associated with bacterial physiology, including stress-response-promoting fitness, antibiotic persistence, and, therefore, general bacterial pathogenesis [13,14,15].

Some studies demonstrated the activation of type II TA systems in stress conditions, like the RelBE system in *Escherichia coli* K-12 with a robust induction of *relBE* transcription under nutritional stress, where RelE reduced the overall translation rate during nutrient scarcity favoring bacterial survival [16]. The antitoxin MqsA was described to be part of the stress response in *E. coli*, wherein the antitoxin represses stress response regulators. When this antitoxin is degraded under specific conditions, the regulator genes are derepressed, enabling the proper response, which can induce other activities such as biofilm formation and reduced motility [15]. In an Uropathogenic *E. coli* (UPEC) strain, one of the main pathogens causing urinary tract infections, the association of the type II TA system PasT-PasI with stress response was demonstrated with an increase in toxin expression, resulting in resistance to nitrosative and oxidative stress, regulating bacterial growth. These studies demonstrate that type II TA systems may be involved in some way with the stress response of bacterial pathogens; consequently, they can often survive in diverse niches by sensing and adapting to changes in environmental cues and altering their gene expression patterns [17].

These challenging environmental conditions may be encountered by hybrid *E. coli* strains, which are pathogenic strains with the potential to cause distinct types of infection in intestinal and extraintestinal niches, due to the presence of virulence factors of diarrheagenic *E. coli* (DEC) and extraintestinal *E. coli* (ExPEC). Hybrid strains are considered to be more virulent than other pathogenic *E. coli* due to their genetic machinery that enables the colonization and multiplication in such different conditions as the intestine, blood, meninges, and urinary tract [18]. For instance, the gastrointestinal tract presents low oxygen concentration, differences in nutrient accessibility, and pH levels that vary from acid in the stomach to alkaline in the intestine [19]. On the other hand, the extraintestinal milieu is characterized by expressive alteration in osmolarity, oxygen radicals, and nutrient availability [20,21].

In a previous study, the *E. coli* strain BA1250 isolated from diarrhea was classified as an atypical enteropathogenic *E. coli* (aEPEC) through the presence of *eae*, the absence of *bfpA*, and the capacity to induce the attaching and effacing (A/E) lesion [22]. Subsequently, after the whole genome sequence analyses, assembly, and annotation of its genome, carried out using the SPAdes and Prokka pipelines, BA1250 was characterized as a hybrid pathogenic aEPEC/ExPEC [23]. Phylogenetic analysis showed that this strain is more related to *E. coli* strains with the potential to cause urinary tract infections [24] than to other aEPEC strains. Recently, we also described that BA1250 was able to infect multiple niches in a zebrafish in vivo model, showing its capacity to colonize and persist in the intestine, bloodstream, and pericardial cavity of the host [25]. Therefore, herein, we investigated the activation of chromosomally expressed type II TA systems present in BA1250 (CcdB-CcdA, MazF-MazE, PasT-PasI, YhaV-PrlF, and YoeB-YefM), and their association to a possible enhancement of hybrid *E. coli* fitness under diverse stress conditions.

## 2. Results

### 2.1. Presence of Type II TA Systems in BA1250 Genome

To assess the presence of type II TA systems in the genome of the hybrid strain BA1250, in silico analyses were conducted using the previously assembled BA1250 genome sequence [23]. The toxin–antitoxin database (TADB) is an integrated database that provides comprehensive information of type II TA systems, and it was used as a platform for TA system identification through TAfinder, concurrently with BLAST alignments.

Twenty-six different TA loci were detected in BA1250, with highly conserved sequences for most of the identified systems present in the genome of *E. coli* from different pathotypes, including diarrheagenic and extraintestinal strains (Table 1). The identified systems belonged to types I, II, IV, and V, with the majority (50%) to type II. Among the identified systems, three of them were observed to be present in more than one copy, varying from two to five copies of each gene. To determine if the detected systems were present in the chromosome or in a plasmid, we used two different methods: the sequence contents-aware plasmid peeler (SCAPP), which is a tool able to identify full circular plasmid sequence, and PlasFlow, which uses a neural network to classify the contig sequences into chromosomal, plasmid, and non-classified sequences. According to this methodology, the majority (95.2%) of the described systems were predicted to be located in the BA1250 chromosome except for system SrnC-SrnB, which was predicted to be plasmid-encoded (Table 1).

### 2.2. Stress Conditions Impair E. coli BA1250 Growth

**Figure 1:**
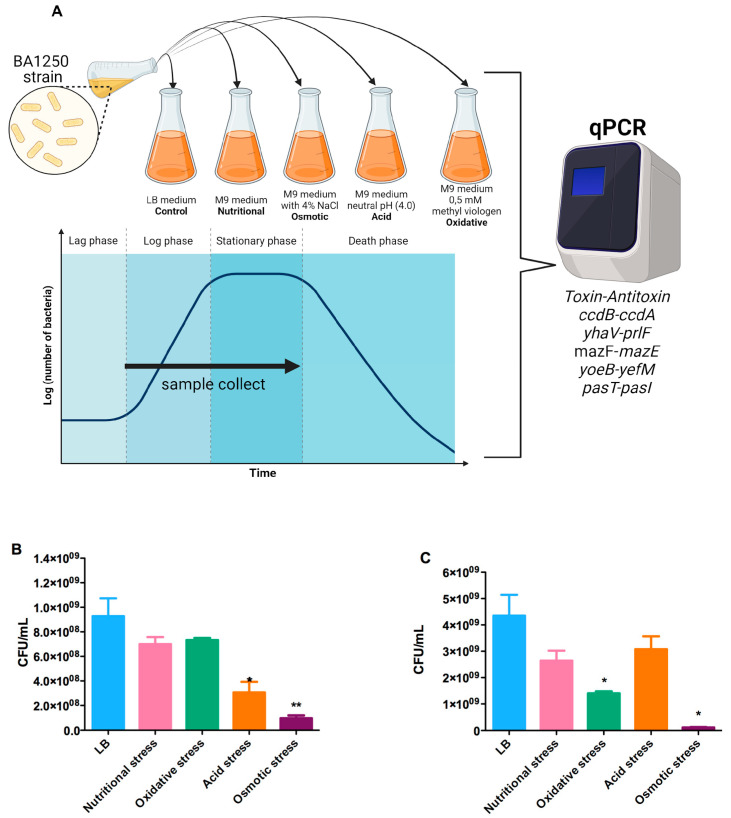
(**A**) Experimental design for bacterial growth under stress. The illustration depicts how the experiments were designed for cultivation under stress conditions, the media employed for bacterial growth, and the bacterial cultivation phase in which samples were collected for qPCR analyses. Created with BioRender.com. Count of BA1250 Colony Forming Units (CFUs/mL) under different culture conditions in the (**B**) logarithmic and (**C**) stationary phase. Statistical analysis was performed using the non-parametric *t*-test, compared to bacteria growth in the LB medium. * *p*-value < 0.02; ** *p*-value < 0.002.

To study the influence of stress factors on TA system activation, whether and how these factors could influence BA1250 growth were initially evaluated. Four types of stress factors were selected according to the possible conditions that a hybrid strain would encounter in the host: nutritional deprivation, osmotic, oxidative, and acid environment. The experimental procedure for the evaluation of stress factors’ influence on bacterial growth is described in Figure 1A, and it was designed to collect samples both in the logarithmic and stationary phases.

Of the conditions studied, there was a significant influence on the growth dynamic between the control condition (LB medium) and osmotic stress, leading to a diminished number of CFUs both in logarithmic and stationary phases (Figure 1B,C). Both acid and oxidative stress factors impacted in a growth-phase-dependent manner, since acid stress altered bacterial growth in the logarithmic phase, leading to a lower number of CFUs, and oxidative stress had no influence. On the other hand, oxidative stress led to a considerable decrease in CFUs in the stationary phase, and acid stress conditions had no disturbance. Surprisingly, there was no significant influence of nutritional deprivation on the number of detected CFUs at either of the studied phases.

### 2.3. TA System Dependent Stress-Response in a Bacterial Logarithmic Growth Phase

To proceed with the study of stress factors in the activation of chromosomal TA systems, five type II TA systems that were detected in only one copy in BA1250 chromosome were selected: CcdA-CcdB, MazE-MazF, PrlF-YhaV, YefM-YoeB, and PasI-PasT. For this reason, BA1250 bacterial samples were obtained from culture in the logarithmic phase under specified stress conditions, and the system’s expression was quantified by qPCR. All qPCR reactions were performed to analyze the relative expression of these genes in comparison to LB cultures.

**Figure 2:**
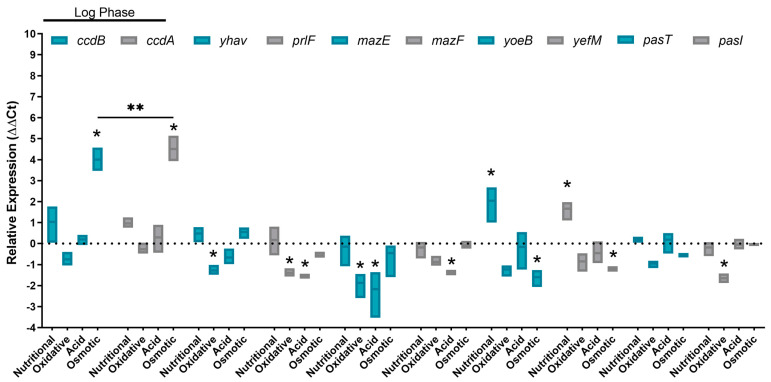
Relative expression of toxin–antitoxins in a log growth phase in duplicates of three independent experiments. *E. coli* BA1250 toxin–antitoxin gene pairs (*ccdB*/*ccdA*, *yhaV/prlF*, *mazE/mazF*, *yoeB/yefM*, and *pasT/pasI*) were evaluated in the log growth phase under nutritional scarcity, oxidative stress, acid shock, osmotic stress, and LB medium condition. Genes were considered up/downregulated when relative average expression was −1 > Log2Fc > 1 in comparison to the LB group. Statistical significance was considered when the *p* value < 0.05 in 2-way ANOVA test comparing toxin and antitoxin at the same condition. (*) Represent statistical significance with *p* < 0.05; (**) represent statistical significance with *p* < 0.01.

In the bacterial logarithmic growth phase, there was a stress-response-dependent system, since the *yoeB-yefM* genes were observed to be upregulated under conditions of nutritional starvation, whereas they were downregulated in the presence of osmotic conditions (Figure 2). The same osmotic condition led to an upregulation in the transcription of the *ccdB-ccdA* genes. The *mazE-mazF* genes were negatively regulated in response to acid stress, while oxidative conditions led to an exclusively downregulation of the antitoxins *mazE* and *pasI.* The contrary was observed for the *yhaV-prlF* genes since they were downregulated under conditions of oxidative stress, but only the antitoxin *prlF* was downregulated in response to acid stress.

### 2.4. Nutritional Starvation and Acid Environment Activate TA Systems in the Bacterial Stationary Growth Phase

**Figure 3:**
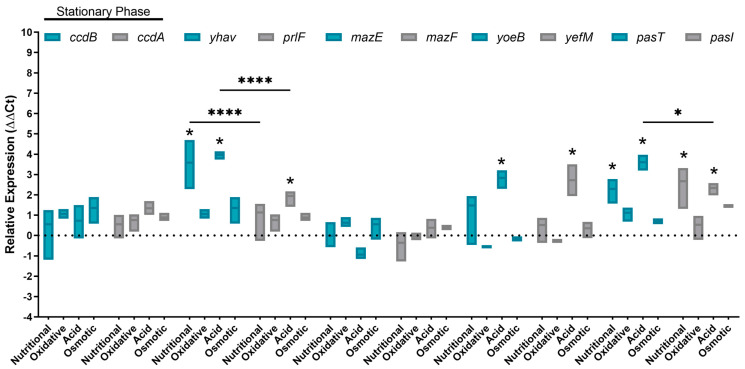
Relative expression of toxin–antitoxins in the stationary growth phase in duplicates of three independent experiments. *E. coli* BA1250 toxin–antitoxin gene pairs (*ccdB/ccdA*, *yhaV/prlF*, *mazE/mazF*, *yoeB/yefM*, and *pasT/pasI*) were evaluated in the stationary growth phase under nutritional scarcity, oxidative stress, acid shock, osmotic stress, and stress-free LB medium condition. Genes were considered up/downregulated when relative average expression was −1 > Log2Fc > 1 in comparison to the LB group. Statistical significance was considered when the *p* value < 0.05 in a 2-way ANOVA test comparing toxin and antitoxin at the same condition. (*) Represent statistical significance with *p* < 0.05; (****) represent statistical significance with *p* < 0.0001.

The assessment of toxin–antitoxin system expression was also performed in the stationary growth phase where in all qPCR reactions the relative expression of the genes was compared to LB cultures. The expression levels of the *ccdB-ccdA* and *mazE-mazF* genes did not exhibit any statistically significant variation under the tested stress conditions during the stationary phase (Figure 3). The same occurred under osmotic and oxidative stresses since it did not activate any of the selected TA systems. In contrast, it was observed that the acid environment had a massive influence on the TA system, since *yhaV-prlF*, *yoeB-yefM*, and *pasT-pasI* genes were activated in this condition. Besides the acid environment, the *past-pasI* genes and the toxin *yhaV* were also active during nutritional starvation.

In specific stress conditions, differences were observed in the expression of only one component of the TA system, either toxin or antitoxin genes. It was evident that most genes in the TA systems were expressed concomitantly. However, notable differences were observed during the log phase for the *yhaV-prlF* genes (in an acid environment) and the *past-pasI*/*mazE-mazF* genes (under oxidative stress). In the stationary phase, a scenario emerged where all systems were expressed similarly, except for the YhaV-PrlF system.

### 2.5. Distribution of Type II TA Systems Among Pathogenic E. coli

Since there was an impressive presence of TA systems in the hybrid strain BA1250, we investigated whether this genetic background would be present in other hybrid *E. coli* strains, as well as in EPEC and ExPEC. Therefore, we selected 53 genomes of EPEC, ExPEC, and hybrid EPEC/ExPEC strains (Figure S1 and Table S1).

The in silico analysis revealed the presence rates of the 21 previously identified TA systems on the BA1250 genome (Table 1), among the studied type II TA systems, i.e., CcdB-CcdA, YhaV-PrlF, MazE-MazF, YoeB-YefM, and PasT-PasI seem to be associated with hybrid strains, as 69.2% of them exhibited all these five TA systems, while 30.8% showed four out of the five (Figure S2).

To characterize the 53 genomes selected, a phylogenetic tree was constructed comparing them to the BA1250 regarding phylogroups (Figure S3). The hybrid strain BA1250 belongs to phylogroup B2, as well as 23 out of the 26 hybrid strains studied, and the remaining three were classified in phylogroup B1. Of the 53 analyzed strains, the majority were distributed between phylogroups B2 (30 strains) and B1 (19 strains), with only 1 strain in phylogroup F and another 3 in phylogroup A (Figure S3).

**Figure 4:**
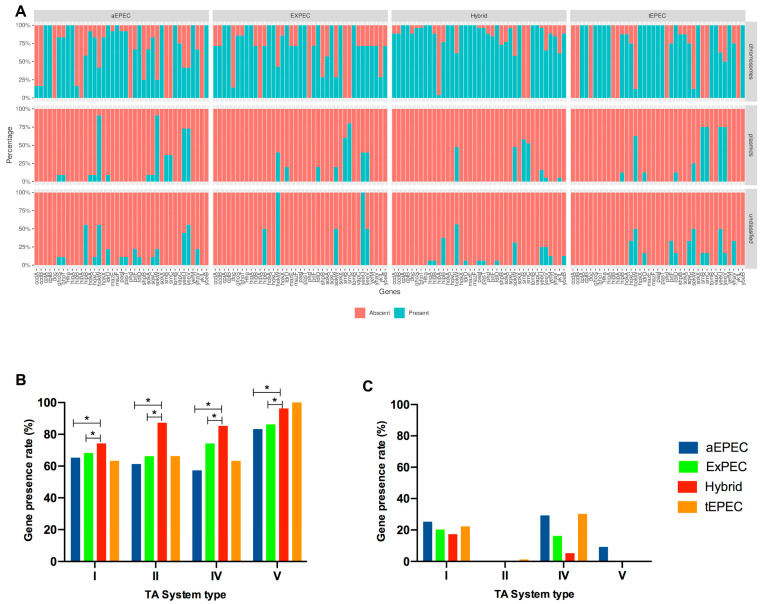
(**A**) Barplot demonstrating the predicted presence and absence of the 39 gene components of TA systems detected into aEPEC, ExPEC, Hybrid, and tEPEC strains chromosomes and plasmids. Bars indicate the percentage of predicted gene presence among the bacterial strains within each pathotype. Gene predicted rate of type I, II, IV, and V TA system components present in aEPEC, ExPEC, Hybrid, and tEPEC strains in the (**B**) chromosome and in the (**C**) plasmid. Bars indicate the percentage of predicted gene presence among the bacterial strains within each TA system type. * *p* < 0.05, as determined by a non-parametric one-way ANOVA test.

The majority of the genes of the analyzed aEPEC, tEPEC, ExPEC, and hybrid ExPEC/EPEC strains were predicted to be located in the chromosome (Figure 4A,B), varying from 53 to 100% presence rate of genes from each type of TA system, with only a small percentage of genes from TA systems type I and IV predicted to be plasmid located in all pathotypes (Figure 4C). However, the predicted presence of type I, II, IV, and V systems in the chromosome was more frequent in the hybrid strains than in the aEPEC strains, indicating a possible gain of function for the first group.

## 3. Discussion

On subsequent investigations built upon the initial findings of TA systems presence in bacteria, hundreds of genomes were examined resulting in the identification of thousands of TA systems within bacterial and archaeal genomes. These studies also unveiled new families of TA systems, and it is now acknowledged that prokaryotic genomes are thoroughly infiltrated by these genes [26]. Among the so far described eight types of TA systems, type II has been extensively researched and is prevalent in bacterial chromosomes, as well as in mobile genetic elements such as plasmids and pathogenicity islands (PAIs).

Several studies have highlighted the substantial presence of these TA genes in various bacterial species. To date, experimental evidence suggests a massive presence of TA systems in pathogenic *E. coli*, but little is known regarding its distribution and function of chromosomal type II TA systems of hybrid *E. coli* strains. In our study, the hybrid strain BA1250 exhibited a considerable number of TA systems within its bacterial genome, since we identified 26 TA loci, with 25 predicted to be located in the chromosome and only 1 in the plasmid. The identified systems were from type I, II, IV, and V, with most type II systems, reinforcing its spread among pathogenic bacteria. Similarly, one study of adherent-invasive *E. coli* (AIEC) NRG857c strain revealed that it encodes at least 33 putative TA systems belonging to types I, II, IV, and V, distributed around all the chromosome, and some close to genomic islands [27].

The abundance of TA genes in the chromosome of pathogenic strains prompts questions about their role, specifically whether these systems play a part in bacterial virulence that provides an advantage during the infectious process. Following the identification of bacterial chromosomes, type II TA systems were initially regarded as advantageous components for adaptation, enhancing the fitness of bacterial populations during stress conditions. This occurs when the toxin is released from the toxin–antitoxin complexes due to antitoxin degradation, allowing the toxin to exert its toxic activity. This activation can result in varied outcomes depending on the model, such as stress responses or antibiotic persistence [28]. These major models were based on studies performed with *E. coli* K-12 laboratory strains, which contain 12 type II systems. However, there is a lack of information regarding the influence of diverse stress environments on the growth dynamics of hybrid *E. coli* strains, as well as how it alters the activation of chromosomal type II TA systems under the same conditions.

The stress conditions frequently associated with TA systems are nutritional scarcity, oxidative and osmotic stress, and acid environments, all of which a hybrid *E. coli* encounters within its host [19,20,21]. Consequently, hybrid strains must adapt to adverse conditions and employ different survival mechanisms. One of the earliest reports linking TA systems to stress responses was provided by Hazan et al. [29], demonstrating that the MazEF system was associated with the response to nutritional stress. Another study with *E. coli* K-12, conducted by Christensen-Dalsgaard and colleagues [30], revealed that the expression of three TA loci was activated during severe nutritional scarcity, and two systems were activated under mild nutritional scarcity. Similarly, in our study, we demonstrated that an environment of nutritional starvation induced the expression of the YoeB-YefM and PasT-PasI systems. Additionally in this condition, there was an induction of only the toxin *yhaV* in the bacterial stationary growth phase, a fact that was also observed in the comprehensive transcription analysis of the *E. coli* O157-H7 CcdB-CcdA system under nutritional scarcity [31]. The explanation of chromosomal type II TA system activation in nutritional scarcity might be linked to bacteria undergoing programmed death, i.e., the cell undergoes a state of growth inhibition, enabling a specific number of cells to endure periods of starvation. This state can be reversed upon encountering nutrient-rich conditions again [28].

In our study, the induction of the type II TA systems under examination do not appear to be linked to the oxidative stress response since there was no upregulation in the expression in any of the selected systems. This result is surprising, given that Hazan et al. [29] demonstrated that the MazEF system triggered cell death in *E. coli* under oxidative stress during the logarithmic phase. On the contrary, the *yhaV-prlF* genes were downregulated in oxidative stress conditions, which corroborates with the findings on pathogenic *Klebsiella pneumoniae*, where the TA system MazEF had its gene expression reduced under oxidative stress [32]. Along with the *yhaV-prlF*, we also observed *mazE* and *pasI* antitoxin downregulation. Frequent upregulation of the toxin in the TA system can impact bacterial virulence. Interestingly, however, the HigA antitoxin within the HigBA TA system in *Pseudomonas aeruginosa* has been found to impair the expression of different virulence genes [33]. A possible explanation for the singular downregulation of the antitoxins here identified could be an effort of the bacteria to counteract the inhibitory role of antitoxins towards virulence factors produced, especially necessary in the diverse stress conditions that a hybrid *E. coli* faces within the host.

There is still no consensus on whether TA systems are regulated independently or in combination with other regulatory factors. However, some evidence points to the notion that some systems may be activated together, as for the MqsA system that was previously linked to the suppression of *rpoS*. Notably, the MqsA antitoxin has been demonstrated to function as a global transcriptional regulator, suppressing the expression of *rpoS* and *csgD* genes that encode pleiotropic regulators involved in stress responses and biofilm formation [15,34]. In a separate study, Bergholz et al. [31] demonstrated that *rpoS* and TA system genes are activated during nutritional stress, coordinating a response that alters metabolic processes and promotes bacterial survival in *E. coli* O157. The transition from exponential to stationary phase modulates the expression of both *rpoS* and TA genes, which may be critical for managing nutritional stress and enabling bacteria to adapt and maintain virulence under adverse conditions. Additionally, another study highlights the connection between TA systems and the stringent response, mediated by guanosine pentaphosphate/tetraphosphate [(p)ppGpp], which activates TA systems during nutrient deprivation. This leads to cell growth inhibition and the formation of persister cells [35].

We also explored the association between type II TA systems with the response to osmotic stress, being the only stress factor that led to a system-dependent regulation demonstrated by a significant upregulation of the CcdB-CcdA system transcription but by a downregulation of the YoeB-YefM system. The CcdB toxin targets DNA gyrase, an essential type II topoisomerase in *E. coli*, blocking polymerase passage and leading to double-strand DNA breaks. Consequently, cellular replication is inhibited, SOS response is induced, and cell filamentation occurs, thus leading to cell death. Therefore, this system seems to be involved in osmotic stress observed by correlation with a decrease in CFU [3,36]. A similar outcome was observed on *E. coli* strains for type II TA system antitoxin genes *mazE* and *hipB*, which were downregulated under NaCl-induced osmotic stress [37]. None of the other systems here studied were influenced by this condition in any of the tested bacterial growth phases.

Special attention must be given to the involvement of the acid environment with the activation of chromosomally expressed type II TA systems in a hybrid *E. coli* since it may activate three out of the five selected systems, namely, *yhaV-prlF, yoeB-yefM,* and *pasTI*. These three systems act on cellular translation and protein synthesis. The PasT toxin specifically targets the 50S ribosomal subunit, while the YoeB and YhaV toxins target mRNA. Therefore, the activation of these systems can influence the decrease in protein synthesis within the cell during acidic stress, consequently favoring cell survival through the induction of dormancy or cellular persistence [38,39,40]. The association between TA systems and UPEC had great importance in the colonization of the niche and the stress response; it was described that during the infectious process of the urinary tract in a mouse model, the PasT-PasI system promoted the colonization and persistence of bacteria in the kidneys, while the TA YefM-YoeB and YbaJ-Hha systems promoted an increase in colonization in the bladder [41]. As our hybrid *E. coli* harbors virulence factors from ExPEC, its capacity to survive and thrive in low pH conditions may facilitate a successful colonization of the urinary tract, a step necessary to cause urinary tract infections [42]. 

Further studies are necessary to precisely determine the specific mechanisms activated within each TA system, as these may vary significantly. In this study, we demonstrated that TA system activation is environment dependent, which may benefit the hybrid strains by enhancing their ability to colonize and replicate in diverse host niches. Our findings also reveal that in the stationary phase, under acid conditions, the BA1250 strain exhibits an increased activation of several systems, underscoring their potential importance in survival and maintenance in this environment. After bacterial invasion, immune cells, such as neutrophils and macrophages, engulf the pathogens, sequestering them within specialized compartments known as phagosomes. Inside these phagosomes, bacteria are subjected to an adverse environment characterized by significant nutrient deprivation, which impairs their survival and leads to cellular death [43,44]. Additionally, proteins associated with innate immunity, such as LPS recognition by TLR4, trigger further responses, including acid stress (via phagosome acidification), thiol, metal, and oxidative stress (through the production of reactive oxygen and nitrogen species, ROS/NOS) [43,44,45,46]. The combination of nutritional deprivation and stress responses creates a hostile environment for invading bacteria, promoting their elimination. Therefore, the PasT-PasI system may act as a virulence factors, enhancing resistance to these conditions, and increasing bacterial survival and pathogenicity. In contrast, the YhaV-PrlF system plays a role in bacteriostasis, and its activation may be an adaptive response to improve bacterial survivability in acid and nutrient-reduced environments.

The massive bacterial genome sequencing revealed that chromosomal toxin–antitoxin systems are widespread, frequently with multiple copies present in a given replicon [4,47], located mostly in genomic islands (prophages, conjugative elements, or transposons) or constitute small genomic islets by themselves [48]. Recent advances in the field suggest alternative views for the roles and functions of these highly abundant and mobile elements in the light of genome evolution. The significant activation of chromosomal type II systems in a condition that would be faced by a hybrid strain led us to investigate the distribution of these systems in a broader range of hybrid, intestinal, and extraintestinal *E. coli* strains.

In this in silico analysis, we observed that of 26 hybrid ExPEC/EPEC strains, the presence of all five type II TA systems was detected in nearly 70% of the analyzed strains, with the remaining 30% exhibiting four out of the five systems. On the other hand, only 14.3% of ExPEC strains and none of the DEC strains presented all the analyzed TA systems. There was a significantly higher presence of TA systems of types I, II, IV, and V in the hybrid strains chromosome than in aEPEC and ExPEC. Although further studies are necessary to comprehend the processes, this might indicate a potential involvement of TA systems, especially type II due to its elevated presence rate, in the physiological processes of hybrid *E. coli* strains.

Our data show how nutritional, osmotic, and acid stresses can positively regulate the transcription of TA genes in a pathogenic hybrid strain, implying that TA systems may become activated and actively participate in the bacterial response to these conditions. Yet, we have shown the wide distribution of TA systems into hybrid *E. coli* strains, especially into the chromosome. Therefore, studies evaluating chromosomal type II TA system expression are necessary for comprehending how significant is the role of these systems in response to stress.

## 4. Conclusions

We have demonstrated the presence of multiple type II TA systems in a hybrid aEPEC/ExPEC strain chromosome and its activation under diverse stress environments. These findings highlight the possible role of the type II TA system in bacterial survival in different niches.

## 5. Materials and Methods

### 5.1. Bacterial Strain

The employed *E. coli* BA1250 strain (serotype O56:H6 by Serotype_finder) [49] was classified as an atypical enteropathogenic *E. coli* (aEPEC) through the presence of *eae*, absence of *bfpA,* and the capacity to induce the A/E lesion on HEp-2 cells [22]. After whole genome sequence analyses, assembly, and annotation of its genome, carried out using the SPAdes and Prokka pipelines, BA1250 was characterized as a hybrid pathogenic aEPEC/ExPEC harboring *eae*, *fyuA*, *yfcV*, *chuA*, *vat*, *tsh*, *hly*E, *sfaH*, *focC*, *fimA*, *papC,* and *astA* genes. [23]. Herein, we performed a phylogenetic analysis employing BlastN (Basic Local Alignment Search Tool) in which the genome was analyzed by kSNP3.0 [50] showing the presence of *eae*, *fyuA*, *yfcV*, *chuA*, and *fimA* genes. Therefore, BA1250 was confirmed as a hybrid aEPEC/ExPEC through the definition of Spurbeck et al. [24]. The strain was stored at −80 °C in Lysogeny broth (LB) with 25% glycerol and was routinely grown in LB for 18 h at 37 °C.

### 5.2. Identification of Type II Toxin–Antitoxin Systems

TAfinder tool was employed, which is an online tool that uses a toxin–antitoxin database (TADB) built through published experimental analyses [51].

Since the TA system genes can be distributed in both the chromosome and plasmid [3], an analysis of the location of the TA genes *sokC*, *hokC*, *sokW*, *hokW*, *srnC*, *srnB*, *sokX*, *hokX*, *rdlD*, *ldrD*, *sokA*, *hokA*, *higA*, *higB*, *vapC, tomb*, *Hha*, *shpB*, *doc*, *ccdA*, *ccdB*, *prlF*, *yhaV*, *phd*, *doc*, *yefM*, *yoeB*, *hipB*, *hipA*, *pasI*, *pasT*, *mazE*, *mazF*, *cptB*, *cptA*, *yeeU*, *yeeV*, *yeeU*, *ykfI*, *ghoS*, and *ghoT* was performed using two methods: plasmid assembly by the algorithm SCAPP [52] and classification of each original assembly contigs in plasmid or chromosomal using neural network of PlasFlow program [53]. To identify TA sequences in either the chromosome or the plasmid, BlastN (Basic Local Alignment Search Tool) was utilized.

### 5.3. Type II TA Systems Expression

#### 5.3.1. Bacterial Growth Conditions

Bacterial culture in LB medium was employed as a control of the “non-stress” growth condition. For all stress conditions used growth experiments and further analyses, bacterial cultivation was performed in M9 medium (6.4 g/L Na_2_HPO_4_-7H_2_O, 1.5 g/L KH_2_PO_4_, 0.25 g/L NaCl, 0.5 g/L NH_4_Cl medium supplemented with 0.1% casamino acids, 0.4% glucose, 2 mM MgSO_4_, and 0.1 mM CaCl_2_) at 37 °C for 16–18 h (nutritional starvation condition). For the osmotic stress, the strain was grown in M9 medium with 4% NaCl; for low pH experiments, cells were washed in M9, adjusted to pH of 4, and cultivated in M9 neutral pH [54]; for the oxidative stress, the culture was performed in LB medium with methyl viologen (0.5 mM) [41].

#### 5.3.2. Colony Forming Unit (CFU) Counting

To quantify bacterial growth under different stress conditions, colony-forming unit (CFU) counting was performed. Bacterial strains were grown in LB medium or minimal M9 medium, depending on the applied stress condition for 16–18 h at 37 °C. The culture was diluted in sterile 0.01 M PBS with a pH of 7.2. Serial dilutions of this material were plated on LB agar for CFU counting. After plating, the plates were incubated at 37 °C for 16 h, and the colonies were subsequently counted. For the logarithmic phase, it was considered an OD_600nm_ of 0.6, and, for the stationary growth phase, it was considered the time where there was no alteration in the OD_600nm_ for more than 30 min.

#### 5.3.3. RNA Extraction and cDNA Synthesis

The RNA was extracted from all samples using the RNeasy mini-kit (Qiagen, Hilden, Germany) according to the manufacturer’s instructions. The RNA obtained was treated with DNase-I to guarantee the absence of DNA in the lysate and to avoid contamination. The RNA obtained from the samples was quantified by NanoDrop 2000 (Thermo Fisher Scientific, Waltham, MA, USA), and 1 µg of RNA was used for the cDNA synthesis reaction. cDNA synthesis was performed using a reverse transcription kit SuperScript III enzyme (Thermo Fisher Scientific, Waltham, MA, USA) according to the manufacturer’s instructions.

#### 5.3.4. Quantitative Real-Time PCR (qRT-PCR)

For qRT-PCR reactions were performed in duplicates of three independent experiments. Briefly, cDNA obtained from each experimental condition was mixed with primers and the qPCR was run on a LightCycler 480 real-time PCR system (Roche, Basel, Switzerland). For the PCR reaction, SYBR Green I Master LightCycler 480 II (Roche, Basel, Switzerland) was used following the manufacturer’s instructions. The relative gene expression (fold change) of TA genes was calculated using the ∆∆Ct method, described by Livak and Schmittgen [55], using *rpoA* and *hcaT* as reference genes. Table 2 shows the sequence of the primers used.

### 5.4. In Silico Analyses of Type II TA Systems in Pathogenic E. coli Strains

To determine the distribution of type II TA systems in aEPEC, tEPEC, ExPEC, and hybrid EPEC/ExPEC strains, in silico analyses were conducted on 53 bacterial genomes deposited in the NCBI database, including our prototype strain BA1250. The bacterial genomes used are described in Table S3. For the selection of hybrid strains, bacteria were chosen based on the presence of *eae* and *bfp* genes as EPEC genetic markers and the presence of *fyuA*, in conjunction with at least two other genes among *vat*, *chuA*, and *yfcV*, as the definition of UPEC [24]. In addition, the genes defined by Johnson et al. [56] to determine the intrinsic virulence of ExPEC (*papA* and/or *papC*, *afa/dra*, *iutA*, and *kpsM*) were employed.

Sequences of genes used to identify hybrid strains (*eae*, *vat*, *fyuA*, *chuA*, *yfcV*, *iucD*, *iutA*, *kpsM*, *papA*, *papC*, *sfaB*, *sfaC*, *sfaD*, *sfaE*, *sfaF*, *sfaG*, *sfaH*, *sfaX*, *sfaY*, *afaA*, *afaB*, *afaC*, *afaD*, *afaE*, *bfpA*, *bfpB*, *bfpC*, *bfpD*, *bfpE*, *bfpF*, *bfpG*, *bfpH*, *bfpI*, *bfpJ*, *bfpK*, and *bfpL*) and to detect the five type II TA systems (*ccdB-ccdA*, *mazF-mazE*, *pasT-pasI*, *yhaV-prlF*, and *yoeB-yefM*) were further analyzed. The analysis to identify similar TA nucleotide sequences and virulence genes in our genomic database was performed using BlastN. Identification of homologous genes was considered for hits with more than 80% identity and 80% coverage. Data were presented as a heatmap and a hierarchical cluster, carried out in the R environment. To construct the phylogenetic tree of the analyzed genomes, kSNP3.0 was utilized [50].

### 5.5. Statistical Analyses

The statistical analyses were performed using the GraphPad Prism 6 software. In the case of CFU analyses, a non-parametric *t*-test was employed. Genes were considered up- or downregulated if the average expression differed by at least 1 Log2 in the ΔΔCt analysis. Statistical significance between genes was determined using a two-way ANOVA, with a threshold of *p* < 0.05.

## Figures and Tables

**Table 1:** Toxin–antitoxin system components are present in the BA1250 genome.

Gene	Function	Type	Number of Copies	Location ^a^
*sokC*	Antitoxin	I	1	Chromosome
*hokC*	Toxin		1	Chromosome
*sokW*	Antitoxin	I	1	Chromosome
*hokW*	Toxin		1	Chromosome
*srnC*	Antitoxin	I	1	Plasmid
*srnB*	Toxin		1	Plasmid
*sokX*	Antitoxin	I	1	Chromosome
*hokX*	Toxin		1	Chromosome
*rdlD*	Antitoxin	I	3	Chromosome
*ldrD*	Toxin		3	Chromosome
*sokA*	Antitoxin	I	1	Chromosome
*hokA*	Toxin		1	Chromosome
*higA*	Antitoxin	II	5	Chromosome
*higB*	Toxin		3	Chromosome
*higA*	Antitoxin	II	5	Chromosome
*vapC*	Toxin		1	Chromosome
*tomB*	Antitoxin	II	1	Chromosome
*Hha*	Toxin		1	Chromosome
*shpB*	Antitoxin	II	1	Chromosome
*doc*	Toxin		2	Chromosome
*ccdA*	Antitoxin	II	1	Chromosome
*ccdB*	Toxin		1	Chromosome
*prlF*	Antitoxin	II	1	Chromosome
*yhaV*	Toxin		1	Chromosome
*phd*	Antitoxin	II	1	Chromosome
*doc*	Toxin		2	Chromosome
*yefM*	Antitoxin	II	1	Chromosome
*yoeB*	Toxin		1	Chromosome
*hipB*	Antitoxin	II	1	Chromosome
*hipA*	Toxin		1	Chromosome
*pasI*	Antitoxin	II	1	Chromosome
*pasT*	Toxin		1	Chromosome
*mazE*	Antitoxin	II	1	Chromosome
*mazF*	Toxin		1	Chromosome
*cptB*	Antitoxin	IV	1	Chromosome
*cptA*	Toxin		1	Chromosome
*yeeU*	Antitoxin	IV	2	Chromosome
*yeeV*	Toxin		1	Chromosome
*yeeU*	Antitoxin	IV	2	Chromosome
*ykfI*	Toxin		1	Chromosome
*ghoS*	Antitoxin	V	1	Chromosome
*ghoT*	Toxin		1	Chromosome

^a^ Predicted location determined by plasmid peeler (SCAPP) and PlasFlow.

**Table 2:** Primer sequences of the toxin–antitoxin system genes and their products.

Gene	Primer Sequence	Product
*ccdB*	*F:* GCCGCGTTTCCCTTTGTTAT	215 pb
	*R:* CAAAATTTCGTTCCCAGCGC	
*ccdA*	*F*: GAGTTAGTCAATCGCGCTCG	158 pb
	*R*: CATCCGGTTTCATCAGCCAA	
*yhaV*	*F:* AAAGGGTTAATGGTTGGGCG	214 pb
	*R:* CCTAACGACTTGCCATGACG	
*prlF*	*F:* GGACAAACAACTATCCCCGC	250 pb
	*R:* CAATGTTGACGTCCATGCCA	
*mazF*	*F:* TGTTGTCCTGAGTCCGTTA	197 pb
	*R:* CTGGGGCAACTGTTCCTTTC	
*mazE*	*F*: AAAGCGTTGGGGAAATTCAC	161 pb
	*R*: TGACCAGTTCAGCAAGCGTA	
*yoeB*	*F:* CTGGTCTGAGGAATCATGGGA	171 pb
	*R:* ATAATGCGTCGGGACCAGAA	
*yefM*	*F:* GAAGCGCGTCAGAATTTGTC	174 pb
	*R:* CATCAATCTCCGGGCGTTAG	
*pasT*	*F:* GTTTACAACCCGCAACCAGT	188 pb
	*R:*TAAACACACGACCAAAGGCG	
*pasI*	*F*: GCGACGGTTGAAGAAGCTAT	161 pb
	*R*: TCGGCAATGAGAGGACGATA	
*hcaT*	*F*: GTTGCCGTGGTTGATAGTGG	165 pb
	*R*: ACGGTCATGATGGCGATACT	
*rpoA*	*F*: CGGCACAATCGATCCTGAAG	173 pb
	*R*: AGCGGACAGTCAATTCCAGA	

## Data Availability

The original contributions presented in this study are included in the article/Supplementary Material. Further inquiries can be directed to the corresponding author(s).

## Supplementary Materials

The following supporting information can be downloaded at https://www.mdpi.com/article/10.3390/toxins16110469/s1, Figure S1. Heatmap indicating the presence or absence of virulence genes of EPEC, ExPEC, and hybrid *E. coli* strains; Figure S2. Heatmap indicating the presence or absence of virulence genes of type II toxin–antitoxin genes in EPEC, ExPEC, and hybrid *E. coli* strains; Figure S3. Phylogenetic tree comparing BA1250 and 52 genomes of EPEC, ExPEC, and hybrid *E. coli*; Table S1. Presence of genetic markers for EPEC and ExPEC in BA1250 and 52 genomes of EPEC, ExPEC, and hybrid *E. coli* strains; Table S2. Presence of type II TA genes in BA1250 and 52 genomes of EPEC, ExPEC, and hybrid *E. coli* strains; Table S3. List of genomes deposited at NCBI used to construct heatmaps and phylogenetic trees.
