# Supplementary material for: Chromosomal Type II Toxin–Antitoxin Systems May Enhance Bacterial Fitness of a Hybrid Pathogenic Escherichia coli Strain Under Stress Conditions
Source: Toxins (Basel). 2024 Nov 1;16(11):469. doi: 10.3390/toxins16110469 (PMC11598369; doi:10.3390/toxins16110469)
Supplement: Supplementary file 1 [file toxins-16-00469-s001.zip › toxins-3233411-supplementary.pdf]

Supplementary Materials: Chromosomal Type II Toxin–Antitoxin Systems May Enhance Bacterial Fitness of a Hybrid Pathogenic *Escherichia coli* Strain Under Stress Conditions

Jessika C. A. Silva, Lazaro M. Marques-Neto, Eneas Carvalho, Alejandra M. G. Del Carpio, Camila Henrique, Luciana C. C. Leite, Thais Mitsunari, Waldir P. Elias, Danielle D. Munhoz and Roxane M. F. Piazza

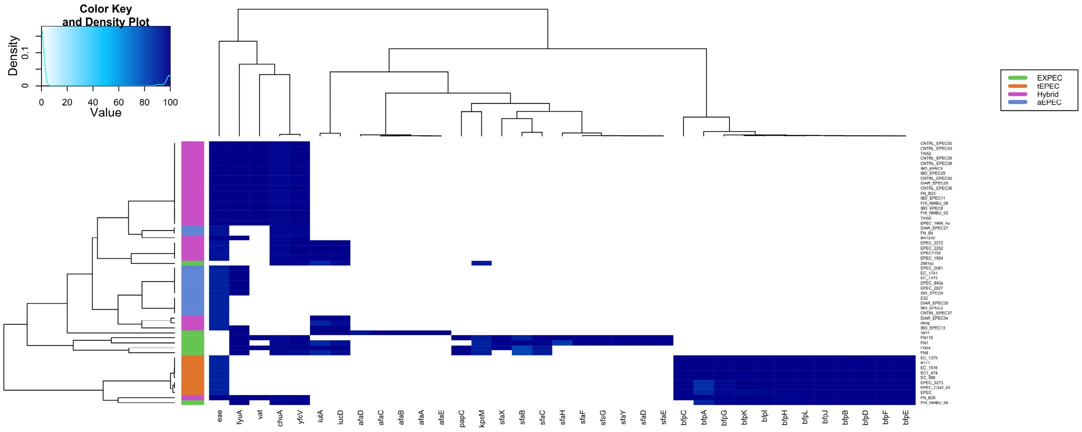

Figure S1. Heatmap indicating presence or absence of virulence genes of EPEC, ExPEC, and hybrid E. coli strains. The figure displays the heatmap of EPEC and ExPEC gene markers to identify hybrid strains by in silico analysis using BlastN carried out in the R environment. Each square, in white or blue, indicates whether a virulence-associated gene is present or absent. The strains are color-coded to reflect their classification as ExPEC, tEPEC, aEPEC, and hybrid E. coli. Additionally, cluster dendrograms are shown, depicting the hierarchical organization of the genomes (on the left) and the genes (at the top), based on presence or absence data.

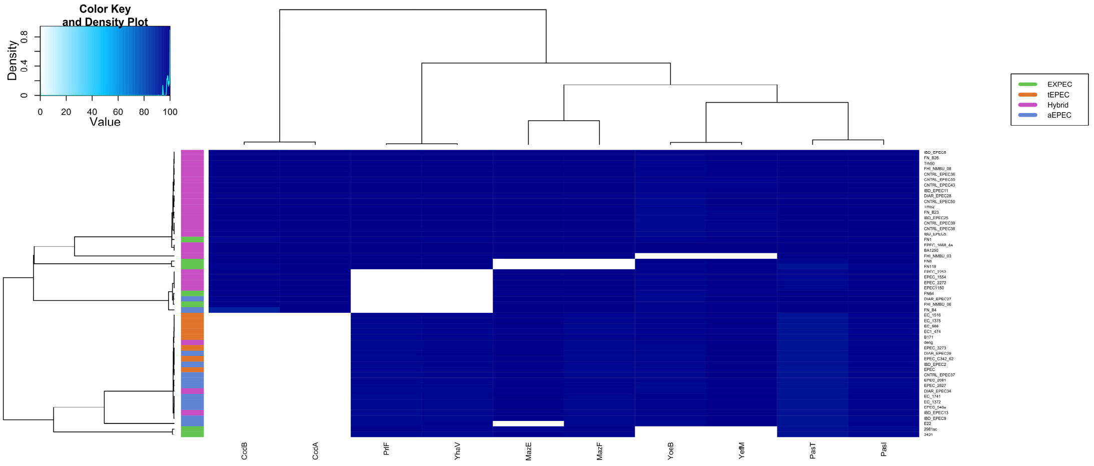

Figure S2. Heatmap indicating presence or absence of virulence genes of type II toxin–antitoxin genes in EPEC, ExPEC, and hybrid E. coli strains. The figure displays the heatmap of type II toxin–antitoxin genes in EPEC, ExPEC, and hybrid strains by in silico analysis using BlastN carried out in the R environment. Each square, in white or blue, indicates whether a virulence-associated gene is present or absent. The strains are color-coded to reflect their classification as ExPEC, tEPEC, aEPEC, and hybrid E. coli. Additionally, cluster dendrograms are shown, depicting the hierarchical organization of the genomes (on the left) and the genes (at the top), based on presence or absence data.

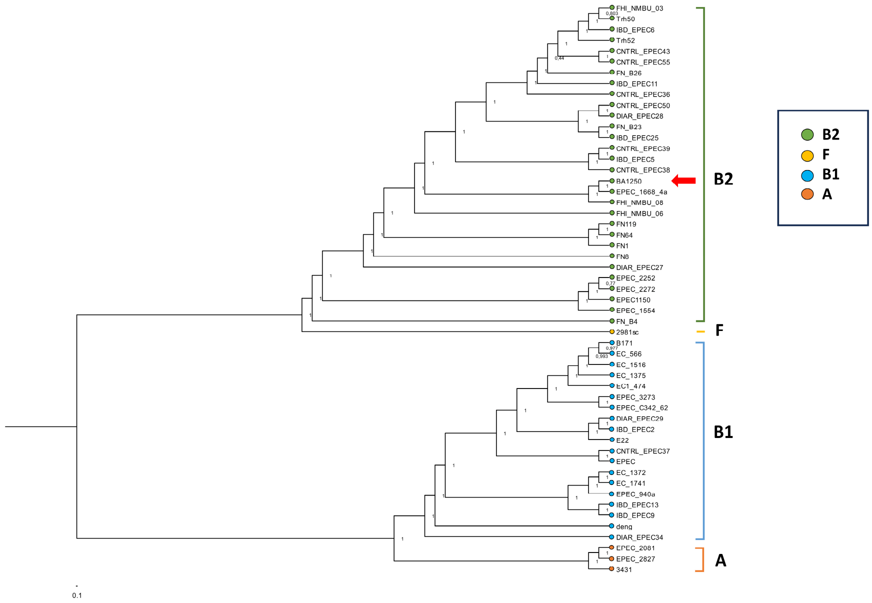

Figure S3. Phylogenetic tree comparing BA1250 and 52 genomes of EPEC, ExPEC, and hybrid E. coli. A phylogram displaying strain BA1250 alongside various reference strains of E. coli (EPEC, ExPEC, and hybrid strains) was created using the KSNP3.0 software for constructing phylogenetic trees, with the maximum likelihood method applied for reconstruction. Branch support was evaluated using the Shimodaira–Hasegawa (SH) test, with lilac dots representing 100% branch reliability. The phylogroups (A, B1, B2, and F) are distinguished by different colors, and the BA1250 strain is marked with a red star.

Table S1. Presence of genetic markers for EPEC and ExPEC in BA1250 and 52 genomes of EPEC, ExPEC, and hybrid E. coli strains.

Table S2. Presence of type II TA genes in BA1250 and 52 genomes of EPEC, ExPEC, and hybrid E. coli strains.

Table S3. List of genomes deposited at NCBI used to construct heatmaps and phylogenetic trees.
